# Efficacy and Safety of a Therapy Combining Sintilimab and Chemotherapy With Cryoablation in the First-Line Treatment of Advanced Nonsquamous Non–Small Cell Lung Cancer: Protocol for a Phase II, Pilot, Single-Arm, Single-Center Study

**DOI:** 10.2196/64950

**Published:** 2024-11-08

**Authors:** Zhiqiang Gao, Jiajun Teng, Rong Qiao, Jialin Qian, Feng Pan, Meili Ma, Jun Lu, Bo Zhang, Tianqing Chu, Hua Zhong

**Affiliations:** 1 Shanghai Chest Hospital Shanghai Jiao Tong University School of Medicine Shanghai China

**Keywords:** cryoablation, immunotherapy, nonsquamous non–small cell lung cancer

## Abstract

**Background:**

Immunotherapy has significantly advanced lung cancer treatment, particularly in nonsquamous non–small cell lung cancer (NSCLC), with overall response rates between 50% and 60%. However, about 30% of patients only achieve a stable disease state. Cryoablation has shown potential to enhance immunotherapy by modifying the tumor’s immune microenvironment through the release of antigens and immune factors. Addressing how to boost the immune response in these patients is critical.

**Objective:**

This study aims to investigate the efficacy and safety of immunochemotherapy in combination with cryoablation as a first-line treatment for advanced NSCLC.

**Methods:**

This is a phase II, pilot, open-label, single arm, single center, interventional study. Patients with stage IIIB to IIIC or IV NSCLC with T staging ranging from T1 to T2b will receive sintilimab (200 mg/m^2^ every 3 weeks) and chemotherapy. After 2 cycles, the feasibility of cryoablation will be considered for those with stable disease by a multidisciplinary team. Cryoablation with 3 freeze-thaw cycles will be performed for the main lesion. The third cycle of systemic therapy will begin 7 (SD 3) days after cryoablation. A total of 20 patients will be enrolled. Treatment will continue until the disease progresses, there is unacceptable toxicity, a participant withdraws consent, other discontinuation criteria are met, or the study reaches completion. The primary objective is to assess progression-free survival (PFS). The secondary objective is to assess efficacy through duration of response, disease control rate, overall survival (OS), and the safety profile. The exploratory objective is to investigate and compare immune factor changes after 2 cycles of immunochemotherapy and at 1, 3, and 7 days after cryoablation. Survival time will be estimated using the Kaplan-Meier method to calculate median PFS and OS. Any adverse events that occur during the trial will be promptly recorded.

**Results:**

The project was funded in 2024, and enrollment will be completed in 2025. The first results are expected to be submitted for publication in 2027.

**Conclusions:**

This study will provide evidence for the efficacy and safety of the combination of immunochemotherapy and cryoablation as a first-line treatment for advanced NSCLC. Although it has a limited sample size, the findings of this study will be used in the future to inform the design of a fully powered, 2-arm, larger-scale study.

**Trial Registration:**

ClinicalTrials.gov NCT06483009; https://clinicaltrials.gov/study/NCT06483009

**International Registered Report Identifier (IRRID):**

PRR1-10.2196/64950

## Introduction

Lung cancer remains the most prevalent and lethal cancer worldwide. The 2022 Global Cancer Observatory report cites around 2.5 million new cases and 1.8 million deaths annually, which encompass all types of lung cancer, confirming its status as the deadliest cancer [1]. It is mainly categorized into 2 types: non–small cell lung cancer (NSCLC), which comprises about 85% of cases, and small-cell lung cancer. In recent years, significant breakthroughs have been made in targeted therapies and immunotherapies. Concurrently, the combination of local and systemic treatments has gained attention. According to the National Comprehensive Cancer Network (NCCN) guidelines [2], for NSCLC patients with oligoprogression after receiving tyrosine kinase inhibitors, local therapies can be incorporated alongside ongoing systemic tyrosine kinase inhibitor treatment for disease control. Studies with small sample sizes have confirmed that both oligoprogression and oligometastases benefit from local treatments [3-5]. The American Society for Radiation Oncology guidelines [6] recommend that for stage IV NSCLC patients with ≤5 oligometastases, up-front definitive local treatment of locoregional primary and all oligometastases should be performed after 2 to 3 months of standard systemic therapy. The BRIGHTSTAR (Brigatinib in Treating Patients With Stage IV or Recurrent Non-small Cell Lung Cancer Trial) study of local consolidative therapy [7,8] found that anaplastic lymphoma kinase (ALK)–rearranged mutation in advanced NSCLC patients who received targeted therapy for 8 weeks followed by local consolidative therapy prolonged the 3-year progression-free survival (PFS) rate compared to a consolidative therapy group (66% in a brigatinib group vs 47% in a crizotinib group, both with advanced ALK inhibitor–naive, ALK-positive non–small cell lung cancer). These studies indicate that sequential local treatment following systemic therapy provides an improved prognosis.

For advanced NSCLC without driver gene mutations, immunotherapy—specifically inhibitors targeting programmed death receptor 1 (PD-1) and its ligand (PD-L1)—has significantly improved patient outcomes. The 5-year overall survival rate has reached 20%, and even 40% in patients with high PD-L1 expression [9]. As a result, immunotherapy is recommended by the NCCN [2] as a standard first-line treatment for this type of lung cancer and has become a mainstream treatment option in clinical practice. Generally, for nonsquamous NSCLC, the objective response rate (ORR) to immunochemotherapy is around 50% to 60% [10-17], with approximately 30% of patients assessed as having stable disease. Sintilimab is a monoclonal antibody that targets PD-1 on T cells, blocking the PD-1/PD-L1 pathway. In NSCLC, sintilimab has been evaluated in a randomized, controlled phase III trial in China known as Oncology Program by Innovent anti-PD-1–11 [13]. This trial assessed the efficacy and safety of sintilimab combined with pemetrexed and platinum chemotherapy compared to a placebo in treating patients with locally advanced or metastatic nonsquamous NSCLC. The results showed that the combination sintilimab-chemotherapy group had significantly prolonged PFS compared to the placebo group, with a median PFS of 8.9 months versus 5.0 months (hazard ratio 0.482, 95% CI 0.362-0.643; *P*<.001). Additionally, the ORR in the combination sintilimab-chemotherapy group increased from 29.8% to 51.9%. Addressing how to enhance the immune response in these patients is crucial. Thus, the integration of local therapies with immunotherapy is gaining focus. The reasons for using local therapy are to (1) reduce tumor burden and minimize the possibility of metastasis and (2) adjust the immune microenvironment and boost the synergetic effect with immunotherapy.

In the NCCN guidelines, image-guided thermal (IGTA) therapy may be considered for those patients who are deemed “high risk”—those with tumors that are for the most part surgically resectable but rendered medically inoperable due to comorbidities. Recently, several studies have demonstrated that IGTA therapy combined with systemic therapy may improve survival [3,4,18]. Combining IGTA therapy and immunotherapy for a synergetic effect should be promising and deserves further investigation [19]. Cryoablation is a minimally invasive technique that uses extremely low temperatures to destroy cells in target tissues; it is commonly applied in treating various benign and malignant diseases, including tumors. This technique boasts benefits like ease of use, minimal intraoperative bleeding, and reduced postoperative pain. At temperatures below –20°C, most cells succumb, and below –50°C, cell death is nearly universal [20]. Cryoablation can cause extracellular ice crystal formation, leading to solute concentration and an osmotic gradient between the inside and outside of the cell, transferring intracellular water to the extracellular space, disrupting the local metabolic environment [21]. The crystals produced by freezing can generate local shear forces to disrupt the integrity of cell membranes, thereby causing lethal damage to the cells. After tumor cells are killed by cryoablation, the necrotic tumor cells at the lesion site release antigens, organelles, DNA, heat shock proteins, and other substances locally, triggering immune cells (such as dendritic cells) to engulf these substances, present antigens through major histocompatibility complex molecules, and costimulate and activate cytotoxic T cells, thus inducing a systemic antitumor immune response. At the same time, after cryotherapy, the levels of cytokines (interleukin [IL]-1, IL-6, tumor necrosis factor [TNF]-α) in the serum significantly increase, also enhancing the immune response [20,22]. The synergetic effect of cryoablation and PD-1 blockade was explored in both allograft mouse lung cancer models and patients with unresectable NSCLC [23]. Cryoablation significantly increased circulating cluster of differentiation (CD) 8+ T cell subpopulations and proinflammatory cytokines in patients with early-stage NSCLC. In lung cancer cell allograft mouse models, an abscopal growth inhibition of contralateral, nonablated tumors was found. Analysis of single-cell RNA and T cell receptor bulk sequencing data revealed that cryoablation reprogrammed the intratumoral immune microenvironment and increased CD8+ T cell infiltration with a higher effector signature, interferon (IFN) response, and cytolytic activity. Mechanistically, cryoablation promoted an antitumor effect through the cyclic GMP-AMP synthase–stimulator of interferon genes–dependent type I IFN signaling pathway. In some small-sample-size clinical studies, patients who received cryoablation plus immunotherapy showed long short-term efficacy compared to monotherapy [24,25]. These studies suggest that cryoablation, as a form of local treatment, combined with immunotherapy, represents a new research direction. Nevertheless, the timing of sequencing therapy, and populations that can benefit from this approach, are still not clear. Further studies with better study designs and prospective cohorts are warranted. The purpose of the study is to evaluate the efficacy and safety of first-line treatment with sintilimab in combination with platinum-based doublet chemotherapy and cryoablation in patients with advanced nonsquamous NSCLC who have been assessed as having stable disease after 2 cycles of treatment.

## Methods

### Study Setting and Sample

This prospective, single-center, single-arm pilot study will be conducted in Shanghai Chest Hospital from July 2024 to July 2027. In our department, we see 100 to 120 new cases of advanced lung cancer per month, which may help us enroll enough patients for this study.

Eligible patients are all those who meet the inclusion criteria. Patients must have a primary lesion with a maximum diameter of <5 cm that is located in a relatively isolated location, not adjacent to major blood vessels or major structures, and deemed suitable for tumor debulking by ablation. After signing an informed consent form, eligible patients meeting the inclusion criteria are screened.

Patients receive sintilimab treatment combined with chemotherapy for 2 cycles, followed by tumor assessment. Patients assessed as having stable disease undergo cryoablation after the assessment. The systemic therapy continues within 7 (SD 3) days after cryoablation. There is currently no consensus on the timing of ablation and immunotherapy. According to the BRIGHSTAR study experience, implementing local consolidation therapy measures after a period of immunotherapy has been found to be feasible and effective. In a study of early-stage NSCLC, cytokines were found to increase from 3 to 7 days after cryoablation [23]. A phase I/II [26], multicenter, open-label, randomized study designed to evaluate the safety and efficacy of ablation combined with toripalimab versus toripalimab monotherapy in treated, unresectable hepatocellular carcinoma, in the first phase of the study, aimed to determine the superior regimen—ablation followed by immunotherapy on day 3 (regimen D3) or day 14 (regimen D14). The study found that regimen D3 resulted in a higher ORR. A 10-year real-world study [27] demonstrated that the combination of cryoablation and immunotherapy treatment showed good efficacy within 2 to 4 weeks. In another study [5], local treatment was administered 4 to 12 weeks after the end of immunotherapy for safety reasons. Taking into consideration patient compliance, safety, and departmental experience, we decided to perform the third cycle of immunotherapy treatment 7 (SD 3) days after cryoablation.

Immunotherapy combined with chemotherapy continues until the disease progresses, there is intolerable toxicity, a patient withdraws informed consent, a patient initiates another antitumor therapy, a patient dies, or a patient meets other criteria for treatment discontinuation specified in the protocol, whichever occurs first. The maximum treatment duration is 24 months.

### Participants

#### Inclusion and Exclusion Criteria

Textbox 1 shows the inclusion and exclusion criteria.

**Inclusion and exclusion criteria.**

**Inclusion criteria**

- Age ≥18 years or ≤75 years
- Histologically or cytologically confirmed locally advanced (stage IIIB to IIIC), metastatic, or recurrent (stage IV) nonsquamous non–small cell lung cancer (NSCLC) (as per the
  International Association for the Study of Lung Cancer and the American Joint Committee on Cancer, 9th edition, tumor, nodes, metastasis lung cancer staging), with T staging ranging from T1 to T2b and the tumor not amenable to surgical treatment and definitive concurrent chemoradiotherapy, as well as not previously having received systemic treatment
- Maximum diameter of the primary lesion <5 cm and located in a relatively isolated area, not adjacent to major blood vessels or major structures
- Absence of epidermal growth factor receptor gene sensitizing mutations, and anaplastic lymphoma kinase gene fusion mutations confirmed with histopathology specimens
- At least 1 measurable lesion according to Response Evaluation Criteria in Solid Tumors (RECIST 1.1), with lesions located within previously irradiated fields considered measurable if confirmed to have progressed
- No prior systemic antitumor therapy for advanced/metastatic disease. Patients who have previously received platinum-based adjuvant/neoadjuvant chemotherapy, or definitive chemoradiotherapy for advanced disease, may be included if disease progression or recurrence has occurred at least 6 months after the end of the last chemotherapy regimen
- Patients with asymptomatic or stable symptomatic brain metastases may be included if they meet specific conditions: (1) measurable lesions outside the central nervous system, (2) absence of central nervous system symptoms or stable symptoms for at least 2 weeks, and (3) no need for corticosteroid treatment, discontinuation of corticosteroid treatment within 7 days before the first dose of study drug, or stable corticosteroid dose reduced to ≤10 mg/day prednisone (or equivalent) within 7 days before the first dose of the study drug
- Eastern Cooperative Oncology Group performance status of 0 to 1
- Expected survival >3 months
- Adequate organ function, with the following laboratory criteria: (1) absolute neutrophil count ≥1.5 × 109/L without granulocyte colony–stimulating factor use in the past 14 days and (2) platelet count ≥100 × 109/L without blood transfusion in the past 14 days

**Exclusion criteria**

- Pathologically confirmed small-cell lung cancer (SCLC), including lung cancer mixed with SCLC and NSCLC
- Diagnosed with malignancies other than NSCLC within 5 years before the first dose of the study drug (excluding cured basal cell carcinoma, squamous cell carcinoma of the skin, and/or in situ carcinoma)
- Previously having received therapy, including anti–programmed death receptor 1, anti–programmed death ligand 1, or anti–programmed death ligand 2, drugs or drugs targeting another T cell receptor for stimulation or coinhibition (eg, cytotoxic T-lymphocyte-associated protein 4, cluster of differentiation [CD] 134, or CD137)
- Active autoimmune diseases requiring systemic treatment within 2 years before the first dose of the study drug; alternative therapies (eg, thyroid hormone, insulin, or physiological corticosteroids for adrenal or pituitary insufficiency) are not considered systemic treatment
- Pregnant or lactating women
- Presence of any severe or uncontrollable systemic diseases
- Not fully recovered from any toxicity and/or complications caused by previous interventions before starting treatment
- A known history of HIV infection (ie, positive for HIV 1/2 antibodies)
- Untreated active hepatitis B (defined as positive hepatitis B surface antigen with hepatitis B virus DNA copy number exceeding the upper limit of normal values for the testing laboratory at the study center) or active hepatitis C virus infection (ie, positive for hepatitis C virus antibodies and hepatitis C virus RNA levels above the lower detection limit)
- Diffuse lesions in both lungs where ablation therapy cannot improve the condition
- Extensive pleural metastases with large amounts of pleural effusion
- Difficulty in needle puncture due to proximity of the tumor to major mediastinal vessels or difficulty in selecting a puncture path due to contrast agent allergy or patient noncompliance
- Severe impairment of lung function, with maximum ventilation <40%

#### Sample Size and Study Duration

The primary objective of this study is to evaluate PFS after adding cryoablation for tumors assessed as stable after 2 cycles of first-line treatment with sintilimab and platinum-based chemotherapy in driver-negative advanced nonsquamous NSCLC patients. Due to the lack of relevant literature regarding the PFS of advanced NSCLC patients who have undergone 2 cycles of immunotherapy and are assessed as having stable disease, this study is exploratory in nature. Therefore, a fixed sample size of 20 patients is set. The anticipated enrollment period for this study is 12 months, with a follow-up duration of 24 months.

### Intervention

#### Sintilimab

Sintilimab (200 mg) is administered by intravenous infusion on day 1 of each cycle, with each cycle lasting 3 weeks, until disease progression, death, intolerable toxicity, withdrawal of informed consent, initiation of new antitumor treatment, or termination of treatment for other reasons, as specified in the protocol. The maximum treatment duration for sintilimab is 24 months.

#### Chemotherapy Regimen

Pemetrexed in combination with a platinum-based regimen is used. Pemetrexed (500 mg/m^2^) is administered by intravenous infusion on day 1 of each cycle, plus either cisplatin (75 mg/m^2^) or carboplatin (area under the curve 5) administered by intravenous infusion on day 1 of each cycle, every 3 weeks, for a total of 4 to 6 cycles. For patients who have not progressed after 4 to 6 cycles, maintenance therapy with pemetrexed monotherapy is continued at the same dose and schedule as before until disease progression, death, intolerable toxicity, withdrawal of informed consent, initiation of new antitumor treatment, or termination of treatment for other reasons, as specified in the protocol. The maximum treatment duration for pemetrexed is 24 months.

#### Cryoablation

For patients assessed as having stable disease after 2 cycles of medication, 1 session is performed under local anesthesia after assessment. The multidisciplinary team, including investigators and radiologists, evaluates the feasibility of tumor ablation based on the size, location, and proximity to major blood vessels. Criteria for eligible lesions include a maximum diameter <5 cm, a relatively isolated lesion location, and the lesion not being adjacent to major blood vessels or vital structures. Cryoablation is performed using a 17 gauge IceRod Plus 1.5 (Boston Scientific Corporation) cryoablation needle, guided by computed tomography (CT), avoiding nearby anatomical structures using the Visual-ICE cryoablation system (Boston Scientific Corporation).

#### Target Lesion Selection for Ablation

Assessment is conducted by a multidisciplinary team including researchers, ablation surgeons, and radiologists during the screening of eligible cases. The main lesion should be <5 cm. Based on tumor size, location, and vascular and adjacent structures, lesions that are assessed as debulkable are selected for cryoablation. For each patient, only the main lesion will be ablated. Debulking is needed when the tumor size exceeds 3 cm or when curative therapy cannot be performed. The ice ball might not cover the whole lesion, but should cover at least 80% of the tumor volume. The purpose is to reduce the tumor burden while the systemic therapy continues.

The cutoff value of 5 cm was chosen (1) for the generalizability of the study results and (2) because the study population comprises patients at stages IIIb to IV. The main purpose is to reduce the tumor burden and stimulate tumor immunity. Considering the heterogeneity of outcomes with different diameters of ablation, the researchers will strive to maintain a consistent ablation zone for patients.

#### Procedures

Cryoablation was performed with a minimum of 3 freeze-thaw cycles. The times for each phase were as follows: 3 minutes of passive thawing, 7 to 12 minutes of freezing, 5 minutes of passive thawing, and 7 to 12 minutes of freezing followed by active thawing. Each procedure was monitored with noncontrast CT imaging, typically at 3-to-5-minute intervals, to visualize the evolving ablation zone to avoid adjacent anatomical structures.

### Outcomes

#### Primary Objective

The primary objective is to evaluate PFS after integrating cryoablation for tumors assessed as stable after 2 cycles of first-line treatment with sintilimab and platinum-based chemotherapy in driver-negative advanced nonsquamous NSCLC patients.

#### Secondary Objectives

The secondary objectives are to assess the ORR of patients, evaluate the disease control rate (DCR) of patients, assess the duration of response (DoR) of patients, and assess the OS of patients. The safety and tolerability of sintilimab in combination with cryoablation and platinum-based doublet chemotherapy will be recorded, including the incidence of adverse events, serious adverse events, and adverse events/serious adverse events that lead to treatment discontinuation.

The primary and secondary efficacy end points of this study are assessed by the investigators using Response Evaluation Criteria in Solid Tumors (RECIST) version 1.1 and iRECIST. In case of any discrepancies, the investigators will consult a third party. The third party comprises a discussion group of experts in the same department (the team of experts performs teaching rounds every Monday morning).

PFS is defined as the time from ablation to disease progression or death from any cause. ORR is defined as the proportion of patients who have a partial or complete response to therapy. DCR is defined as the proportion of patients who have stable disease or a partial or complete response to therapy. DoR is defined as the time from ablation to disease progression or death in patients who achieve a complete or partial response. OS is defined as the time from ablation to death from any cause.

#### Exploratory Objective

The exploratory objective is a flow cytometry analysis of the peripheral expression of immune factors after 2 cycles of immunotherapy plus chemotherapy and at 1, 3, and 7 days after cryoablation. Peripheral blood samples of 2 ml each are collected. Changes in immune factors, including CD4+ T cells, naive CD8+ T cells (CD45RA+chemokine receptor [CCR] 7+), central memory CD8+ T cells (CD45RA− CCR7+), effector CD8+ T cells (CD45RA+ CCR7−), effector memory CD8+ T cells (CD45RA− CCR7−), IL-2, TNF-β, IFN-γ, IL-4, IL-6, IL-10, IL-11, IL-29, regulatory T cells, and matrix metalloproteinase–3 are explored using flow cytometry.

### Statistical Analysis

All statistical analyses will be performed using SPSS (version 23 or higher; IBM Corp). Two-sided hypothesis tests will be used for all statistical tests, and comparisons between groups will provide 95% CIs and *P* values. Unless otherwise specified, continuous data will be described statistically using the mean (SD) or median (minimum, maximum), while categorical data will be described using frequencies (percentages). Cytokine changes before and after treatment will be tested with repeated ANOVA or the Friedman test as appropriate. Survival time will be estimated using the Kaplan-Meier method to calculate median PFS and OS (with the 95% CI), and survival curves will be plotted.

### Data Management

The raw data will be captured using case report forms (CRFs; these are paper based) at the designated time. One data administrator, who is not affiliated with the research team and is blinded to group allocation, independently receives the completed CRFs and enters the raw data into a Microsoft Excel database. Simultaneously, they will input real-time data into ClinicalTrials.gov.

### Ethical Considerations

Ethical approval to conduct this study has been obtained from the Research Ethics Committee of Shanghai Chest Hospital (IS24069) on June 28, 2024. Any substantial amendment to the study protocol will need to be approved by an ethics committee after being approved by the sponsor. Informed consent is obtained from participants and their ability to opt out is communicated to them. The data of patients are anonymized or deidentified. To ensure privacy and confidentiality, all records and files related to the study are kept in a secure location and are only accessible to members of the research team. The results will be published in peer-reviewed journals and presented at relevant national and international conferences. The study findings will also be disseminated through presentations at relevant academic forums.

## Results

The project was funded in 2024, and enrollment will be completed in 2025. The first results are expected to be submitted for publication in 2027.

## Discussion

### Overview

Immunotherapy has established its position as a first-line treatment for NSCLC, with some patients still being assessed as having stable disease after a period of treatment. Nevertheless, several studies have confirmed that combining cryoablation with systemic treatments yields better outcomes. Niu et al [25] explored the efficacy of cryoablation treatment compared to palliative treatment in stage IV lung cancer. In their study, 98 lesions in 31 patients received cryoablation treatment, while 23 patients received palliative treatment. The results showed that the OS in the cryoablation group was significantly better than in the palliative treatment group (median OS: 14 months vs 7 months, *P*≤.001). Multiple cryoablation treatments were found to improve OS more than a single cryoablation treatment (median OS: 18 months vs 14 months, *P*=.04). Another study [24] included 64 patients, including 32 patients who received argon-helium knife cryoablation combined with nivolumab treatment (the cryoablation-nivolumab group) and 32 patients who received cryoablation treatment alone (the cryoablation group). The results showed that short-term efficacy was better in the cryoablation-nivolumab group than the cryoablation group, with a statistically significant difference (*P*<.05). The DCR in the cryoablation-nivolumab group was 87.5%, compared to 62.5% in the cryoablation group (*P*=.02). The total number of T cells, CD8+ T cells, and CD4+ T cells significantly increased in the cryoablation-nivolumab group (*P*<.05), and both groups showed an increase in NK cell count after treatment (*P*<.05). Regarding lymphocyte function, the levels of IL-2 and TNF-β increased in the cryoablation-nivolumab group (*P*<.05), and the levels of IFN-γ increased in both groups after treatment (*P*<.05). These initial studies confirmed the efficacy of combining cryoablation and immunotherapy, but several questions regarding the combination therapy still need to be answered and clarified: how to maximize the value of the combination therapy, how to find the appropriate timing, and how to identify populations that could benefit. This study will help to clarify the mechanisms and optimal sequential timing of combining cryoablation with immunotherapy. Our study aims to improve the prognosis of patients by reducing the local tumor burden and stimulating the immune response. Should our study yield positive results, we plan to conduct a larger-scale, 2-arm study.

### Principal Findings

This study will provide evidence for the efficacy and safety of the combination of immunochemotherapy and cryoablation as a first-line treatment for advanced NSCLC. Although it has a limited sample size, the findings of this study can be used in the future to inform the design of a fully powered, 2-arm, larger-scale study.

### Limitations

There are risks inherent in the study design and conception. First, our sample size was not rigorously calculated; instead, a fixed sample size of only 20 was chosen, which is relatively small. Second, this is a single-center study, which may introduce bias in the results. Third, this is a single-arm study lacking a control group, which prevents comparative conclusions. These limitations are expected to be addressed in future research.

### Conclusion

In conclusion, our study is pivotal in clarifying the timing of ablation and identifying the populations that might benefit from this treatment, and it lays the foundation for larger future studies.

## Appendix

Multimedia Appendix 1 Peer review report from the Investigator-Sponsored Research Program (ISP) - Boston Scientific.

## Abbreviations

ALK: anaplastic lymphoma kinase
BRIGHTSTAR: Brigatinib in Treating Patients With Stage IV or Recurrent Non-small Cell Lung Cancer Trial
CCR: chemokine receptor
CD: cluster of differentiation
CT: computed tomography
DCR: disease control rate
DoR: duration of response
IFN: interferon
IGTA: image-guided thermal
IL: interleukin
NCCN: National Comprehensive Cancer Network
NSCLC: non–small cell lung cancer
ORR: objective response rate
OS: overall survival
PD-1: programmed death receptor 1
PD-L1: programmed death ligand 1
PFS: progression-free survival
RECIST: Response Evaluation Criteria in Solid Tumors
TNF: tumor necrosis factor

## Data Availability

The data sets generated or analyzed during this study are available from the corresponding author on reasonable request.

## References

[1] Bray F, Laversanne M, Sung H, Ferlay J, Siegel R, Soerjomataram I, Jemal Ahmedin (2024). Global cancer statistics 2022: GLOBOCAN estimates of incidence and mortality worldwide for 36 cancers in 185 countries. CA Cancer J Clin.

[2] Riely Gregory J, Wood Douglas E, Ettinger David S, Aisner Dara L, Akerley Wallace, Bauman Jessica R, Bharat Ankit, Bruno Debora S, Chang Joe Y, Chirieac Lucian R, DeCamp Malcolm, Desai Aakash P, Dilling Thomas J, Dowell Jonathan, Durm Gregory A, Gettinger Scott, Grotz Travis E, Gubens Matthew A, Juloori Aditya, Lackner Rudy P, Lanuti Michael, Lin Jules, Loo Billy W, Lovly Christine M, Maldonado Fabien, Massarelli Erminia, Morgensztern Daniel, Mullikin Trey C, Ng Thomas, Owen Dawn, Owen Dwight H, Patel Sandip P, Patil Tejas, Polanco Patricio M, Riess Jonathan, Shapiro Theresa A, Singh Aditi P, Stevenson James, Tam Alda, Tanvetyanon Tawee, Yanagawa Jane, Yang Stephen C, Yau Edwin, Gregory Kristina M, Hang Lisa (2024). Non-Small Cell Lung Cancer, Version 4.2024, NCCN Clinical Practice Guidelines in Oncology. J Natl Compr Canc Netw.

[3] Ni Y, Bi J, Ye X, Fan W, Yu G, Yang X, Huang Guanghui, Li Wenhong, Wang Jiao, Han Xiaoying, Ni Xiang, Wei Zhigang, Han Mingyong, Zheng Aimin, Meng Min, Xue Guoliang, Zhang Liang, Wan Chao (2016). Local microwave ablation with continued EGFR tyrosine kinase inhibitor as a treatment strategy in advanced non-small cell lung cancers that developed extra-central nervous system oligoprogressive disease during EGFR tyrosine kinase inhibitor treatment: A pilot study. Medicine (Baltimore).

[4] Ni Y, Ye X, Yang X, Huang G, Li W, Wang J, Han X, Wei Z, Meng M (2020). Microwave ablation as local consolidative therapy for patients with extracranial oligometastatic EGFR-mutant non-small cell lung cancer without progression after first-line EGFR-TKIs treatment. J Cancer Res Clin Oncol.

[5] Bauml JM, Mick R, Ciunci C, Aggarwal C, Davis C, Evans T, Deshpande C, Miller L, Patel P, Alley E, Knepley C, Mutale F, Cohen RB, Langer CJ (2019). Pembrolizumab after completion of locally ablative therapy for oligometastatic non-small cell lung cancer: a phase 2 trial. JAMA Oncol.

[6] Iyengar P, All S, Berry MF, Boike TP, Bradfield L, Dingemans AC, Feldman J, Gomez DR, Hesketh PJ, Jabbour SK, Jeter M, Josipovic M, Lievens Y, McDonald F, Perez BA, Ricardi U, Ruffini E, De Ruysscher D, Saeed H, Schneider BJ, Senan S, Widder J, Guckenberger M (2023). Treatment of oligometastatic non-small cell lung cancer: an ASTRO/ESTRO clinical practice guideline. Pract Radiat Oncol.

[7] Antonia SJ, Villegas A, Daniel D, Vicente D, Murakami S, Hui R, Kurata T, Chiappori A, Lee KH, de Wit M, Cho BC, Bourhaba M, Quantin X, Tokito T, Mekhail T, Planchard D, Kim Y, Karapetis CS, Hiret S, Ostoros G, Kubota K, Gray JE, Paz-Ares L, de Castro Carpeño Javier, Faivre-Finn C, Reck M, Vansteenkiste J, Spigel DR, Wadsworth C, Melillo G, Taboada M, Dennis PA, Özgüroğlu Mustafa, PACIFIC Investigators (2018). Overall survival with durvalumab after chemoradiotherapy in stage III NSCLC. N Engl J Med.

[8] Antonia Scott J, Villegas Augusto, Daniel Davey, Vicente David, Murakami Shuji, Hui Rina, Yokoi Takashi, Chiappori Alberto, Lee Ki H, de Wit Maike, Cho Byoung C, Bourhaba Maryam, Quantin Xavier, Tokito Takaaki, Mekhail Tarek, Planchard David, Kim Young-Chul, Karapetis Christos S, Hiret Sandrine, Ostoros Gyula, Kubota Kaoru, Gray Jhanelle E, Paz-Ares Luis, de Castro Carpeño Javier, Wadsworth Catherine, Melillo Giovanni, Jiang Haiyi, Huang Yifan, Dennis Phillip A, Özgüroğlu Mustafa, PACIFIC Investigators (2017). Durvalumab after chemoradiotherapy in stage III non-small-cell lung cancer. N Engl J Med.

[9] Garon EB, Hellmann MD, Rizvi NA, Carcereny E, Leighl NB, Ahn M, Eder JP, Balmanoukian AS, Aggarwal C, Horn L, Patnaik A, Gubens M, Ramalingam SS, Felip E, Goldman JW, Scalzo C, Jensen E, Kush DA, Hui R (2019). Five-year overall survival for patients with advanced non‒small-cell lung cancer treated with pembrolizumab: results from the phase I KEYNOTE-001 study. J Clin Oncol.

[10] Gadgeel S, Rodríguez-Abreu Delvys, Speranza G, Esteban E, Felip E, Dómine Manuel, Hui R, Hochmair MJ, Clingan P, Powell SF, Cheng SY, Bischoff HG, Peled N, Grossi F, Jennens RR, Reck M, Garon EB, Novello S, Rubio-Viqueira B, Boyer M, Kurata T, Gray JE, Yang J, Bas T, Pietanza MC, Garassino MC (2020). Updated analysis from KEYNOTE-189: pembrolizumab or placebo plus pemetrexed and platinum for previously untreated metastatic nonsquamous non-small-cell lung cancer. J Clin Oncol.

[11] Gray J, Rodríguez-Abreu D, Powell S, Hochmair M, Gadgeel S, Esteban E, Felip E, Speranza G, De Angelis F, Dómine M, Cheng S, Bischoff H, Peled N, Reck M, Hui R, Garon E, Boyer M, Kurata T, Yang J, Jensen E, Souza F, Garassino M (2021). FP13.02 pembrolizumab + pemetrexed-platinum vs pemetrexed-platinum for metastatic NSCLC: 4-year follow-up from KEYNOTE-189. J Thorac Oncol.

[12] Lu S, Wang J, Yu Y, Yu X, Hu Y, Ai X, Ma Z, Li X, Zhuang W, Liu Y, Li W, Cui J, Wang D, Liao W, Zhou J, Wang Z, Sun Y, Qiu X, Gao J, Bao Y, Liang L, Wang M (2021). Tislelizumab plus chemotherapy as first-line treatment for locally advanced or metastatic nonsquamous NSCLC (RATIONALE 304): a randomized phase 3 trial. J Thorac Oncol.

[13] Yang Y, Wang Z, Fang J, Yu Q, Han B, Cang S, Chen G, Mei X, Yang Z, Ma R, Bi M, Ren X, Zhou J, Li B, Song Y, Feng J, Li J, He Z, Zhou R, Li W, Lu Y, Wang Y, Wang L, Yang N, Zhang Y, Yu Z, Zhao Y, Xie C, Cheng Y, Zhou H, Wang S, Zhu D, Zhang W, Zhang L (2020). Efficacy and safety of sintilimab plus pemetrexed and platinum as first-line treatment for locally advanced or metastatic nonsquamous NSCLC: a randomized, double-blind, phase 3 study (Oncology pRogram by InnovENT anti-PD-1-11). J Thorac Oncol.

[14] Zhou C, Chen G, Huang Y, Zhou J, Lin L, Feng J, Wang Z, Shu Y, Shi J, Hu Y, Wang Q, Cheng Y, Wu F, Chen J, Lin X, Wang Y, Huang J, Cui J, Cao L, Liu Y, Zhang Y, Pan Y, Zhao J, Wang L, Chang J, Chen Q, Ren X, Zhang W, Fan Y, He Z, Fang J, Gu K, Dong X, Jin F, Gao H, An G, Ding C, Jiang X, Xiong J, Zhou X, Hu S, Lu P, Liu A, Guo S, Huang J, Zhu C, Zhao J, Gao B, Chen Y, Hu C, Zhang J, Zhang H, Zhao H, Zhou Y, Tai Y (2021). P79.02 updated OS and time to second progression with first-line camrelizumab plus chemo vs chemo for advanced non-squamous NSCLC. J Thorac Oncol.

[15] Zhou C, Chen G, Huang Y, Zhou J, Lin L, Feng J, Wang Z, Shu Y, Shi J, Hu Y, Wang Q, Cheng Y, Wu F, Chen J, Lin X, Wang Y, Huang J, Cui J, Cao L, Liu Y, Zhang Y, Pan Y, Zhao J, Wang L, Chang J, Chen Q, Ren X, Zhang W, Fan Y, He Z, Fang J, Gu K, Dong X, Zhang T, Shi W, Zou J, CameL Study Group (2021). Camrelizumab plus carboplatin and pemetrexed versus chemotherapy alone in chemotherapy-naive patients with advanced non-squamous non-small-cell lung cancer (CameL): a randomised, open-label, multicentre, phase 3 trial. Lancet Respir Med.

[16] Zhou C, Wang Z, Sun Y, Cao L, Ma Z, Wu R, Yu Y, Yao W, Chang J, Chen J, Zhuang W, Cui J, Chen X, Lu Y, Shen H, Li P, Wang J, Sun B, Lu D, Yang J (2020). LBA4 GEMSTONE-302: A phase III study of platinum-based chemotherapy (chemo) with placebo or CS1001, an anti-PDL1 antibody, for first-line (1L) advanced non-small cell lung cancer (NSCLC). Ann Oncol.

[17] West H, McCleod M, Hussein M, Morabito A, Rittmeyer A, Conter HJ, Kopp H, Daniel D, McCune S, Mekhail T, Zer A, Reinmuth N, Sadiq A, Sandler A, Lin W, Ochi Lohmann T, Archer V, Wang L, Kowanetz M, Cappuzzo F (2019). Atezolizumab in combination with carboplatin plus nab-paclitaxel chemotherapy compared with chemotherapy alone as first-line treatment for metastatic non-squamous non-small-cell lung cancer (IMpower130): a multicentre, randomised, open-label, phase 3 trial. Lancet Oncol.

[18] Yang H, Li M, Mei T (2022). Survival benefit of thermal ablation combined with chemotherapy for the treatment of stage IV nonsmall cell lung cancer: a propensity-matched analysis. Int J Hyperthermia.

[19] Palussière Jean, Catena V, Lagarde P, Cousin S, Cabart M, Buy X, Chomy F (2019). Primary tumors of the lung: should we consider thermal ablation as a valid therapeutic option?. Int J Hyperthermia.

[20] Baust J, Gage AA, Ma H, Zhang C (1997). Minimally invasive cryosurgery--technological advances. Cryobiology.

[21] Felsenstein KM, Theodorescu D (2018). Precision medicine for urothelial bladder cancer: update on tumour genomics and immunotherapy. Nat Rev Urol.

[22] Abdo J, Cornell DL, Mittal SK, Agrawal DK (2018). Immunotherapy plus cryotherapy: potential augmented abscopal effect for advanced cancers. Front Oncol.

[23] Gu C, Wang X, Wang K, Xie F, Chen L, Ji H, Sun J (2024). Cryoablation triggers type I interferon-dependent antitumor immunity and potentiates immunotherapy efficacy in lung cancer. J Immunother Cancer.

[24] Feng J, Guiyu D, Xiongwen W (2021). The clinical efficacy of argon-helium knife cryoablation combined with nivolumab in the treatment of advanced non-small cell lung cancer. Cryobiology.

[25] Niu L, Chen J, Yao F, Zhou L, Zhang C, Wen W, Bi X, Hu Y, Piao X, Jiang F, Zeng J, Liu W, Li J, He L, Mu F, Zuo J, Xu K (2013). Percutaneous cryoablation for stage IV lung cancer: a retrospective analysis. Cryobiology.

[26] Zhou C, Li Y, Li J, Song B, Li H, Liang B, Gu Shanzhi, Li Haiping, Chen Changyong, Li Sai, Peng Changli, Liu Fei, Xiao Juxiong, Long Xueying, Li Ping, Xiong Zhengping, Yi Xiaoping, Liao Weihua, Shi Liangrong (2023). A phase 1/2 multicenter randomized trial of local ablation plus toripalimab versus toripalimab alone for previously treated unresectable hepatocellular carcinoma. Clin Cancer Res.

[27] Bonnet B, Tournier L, Deschamps F, Yevich S, Marabelle A, Robert C, Albiges L, Besse B, Bonnet V, De Baère Thierry, Tselikas L (2024). Thermal ablation combined with immune checkpoint blockers: a 10-year monocentric experience. Cancers (Basel).

