# Supplementary material for: Efficacy and Safety of a Therapy Combining Sintilimab and Chemotherapy With Cryoablation in the First-Line Treatment of Advanced Nonsquamous Non–Small Cell Lung Cancer: Protocol for a Phase II, Pilot, Single-Arm, Single-Center Study
Source: JMIR Res Protoc. 2024 Nov 8;13:e64950. doi: 10.2196/64950 (PMC11584530; doi:10.2196/64950)
Supplement: Multimedia Appendix 1 [file resprot_v13i1e64950_app1.pdf]

1. Protocol states both mRECIST and RECIST will be used – need clarification.

Due to the design of this study involves tumor immunotherapy efficacy evaluation, after discussions with the investigators, it was decided to use iRECIST and RECIST V1.1 criteria as references. In case of any disagreements, a third party will be consulted.

The third party refers to the discussion group of experts in the same department (the expert teaching rounds team every Monday morning)

The amendment was as follows:

V1.0	V1.1
Secondary Objectives:<br><br>Assess the objective response rate (ORR) of subjects according to mRECIST V1.1.<br><br>Evaluate the disease control rate (DCR) of subjects according to mRECIST V1.1.<br><br>Assess the duration of response (DOR) of subjects according to mRECIST V1.1.	Secondary Objectives:<br><br>Assess the objective response rate (ORR) of subjects determined by investigators<br><br>Evaluate the disease control rate (DCR) of subjects determined by investigators<br><br>Assess the duration of response (DOR) of subjects determined by investigators<br><br>The primary and secondary efficacy endpoints of this study are assessed by the investigators using the Response Evaluation Criteria in Solid Tumors (RECIST)1.1 and iRECIST. In case of any discrepancies, the investigators will consult a third party.

2. Also require clarification about what 'debulking ablation' is defined as.

Here Debulking is defined for lesions that exceeds 3cm or where the curative therapy cannot be performed.

V1.0	V1.1

Based on tumor size, location, vascular and adjacent structures, lesions that can be reduced by assessment are selected for cryoablation.	Based on tumor size, location, vascular and adjacent structures, lesions that can be debulked by assessment are selected for cryoablation. For each patient, only the main lesion will be ablated. Debulking is needed when the tumor size exceeds 3cm or where the curative therapy cannot be performed. The ice ball maybe not cover the whole lesion but at least 80% of the tumor volume. The purpose is to reduce tumor burden while the systemic therapy continues.

3. Need further information regarding number of lesions, size and how ablated.

We will only ablate the main lesion, which is < 5cm. Lesions suitable for debulking decided by MDT discussion based on tumor size, location, relationship with blood vessels, and adjacent structures are selected for cryoablation. We further added details of procedures of ablation.

V1.0	V1.1
None	Procedures: CA was performed with a minimum of 3 freeze–thaw cycles. The times for each phase are: 3-min freeze, 3-min passive thaw, 7-12 min freeze, 5-min passive thaw, 7-12 min freeze followed by active thawing. Each procedure was monitored with non-contrast CT imaging typically at 3 to 5 minutes intervals to

	visualize the evolving ablation zone to avoid adjacent anatomical structures.

4. Are patients with lesions >5 cm excluded?

Yes, in the inclusion criteria, only patients with T1b will be included. Only the main lesions will be ablated, because the researchers want to see the if the cryotherapy stimulates immune effect.

5. Other amendment

After the assessment of the second cycle and determined as SD, the ablation will be performed. The third systemic treatment cycle will be performed within 7±3days. This is clarified in the protocol, too.

V1.0	V1.1
Patients receive Sintilimab treatment combined with chemotherapy for two cycles, followed by tumor assessment. Patients assessed as having stable disease (SD) undergo cryoablation within 7 days ±3 days after the assessment.	Patients receive Sintilimab treatment combined with chemotherapy for two cycles, followed by tumor assessment. Patients assessed as having stable disease (SD) undergo cryoablation after the assessment. The systemic therapy continues within 7 days ±3 days after cryoablation.
